# Transcatheter Procedure Versus Surgical Interventions for Severe Aortic Stenosis: A Contemporary Evaluation Against Conservative Management

**DOI:** 10.7759/cureus.71859

**Published:** 2024-10-19

**Authors:** Zhiyuan Ma, Shahrad Shadman, Yaniv Maddahi, Mahesh Krishnamurthy, Peter Puleo, Jamshid Shirani

**Affiliations:** 1 Department of Cardiology, St. Luke's University Health Network, Bethlehem, USA; 2 Department of Internal Medicine, St. Luke's University Health Network, Bethlehem, USA

**Keywords:** all-cause mortality, aortic stenosis, conservative therapy, meta-analysis, surgical aortic valve replacement, transcatheter aortic valve replacement

## Abstract

For Aortic valve replacement (AVR), both transcatheter aortic valve replacement (TAVR) and surgical aortic valve replacement (SAVR) serve as a pivotal therapeutic approach for severe aortic stenosis (AS). While both modalities show advantages over conservative management, the long-term mortality benefits post AVR, especially when comparing TAVR with SAVR, remain uncertain. A comprehensive meta-analysis was conducted through a systematic search of electronic databases up to December 7, 2023. Individual patient data extracted from Kaplan-Meier plots underwent pooling and modeling with stratification by surgical risk. The primary endpoint was all-cause mortality at five years. The study included 11 randomized controlled trials (RCTs) and 12 non-RCTs, encompassing 4,215 patients undergoing TAVR, 4,017 undergoing SAVR, and comparing 11,285 AVR patients with 23,358 receiving conservative management. Transcatheter aortic valve replacement exhibited significantly lower all-cause mortality at six months (hazard ratio (HR) 0.62, 95% CI: 0.52-0.74) compared to SAVR, with no significant difference beyond 6 months (HR 1.08, 95% CI: 0.98-1.19). There were no significant differences in cardiovascular mortality (HR 0.98, 95% CI: 0.83-1.16), stroke (HR 1.02, 95% CI: 0.75-1.38), or valvular hemodynamics between TAVR and SAVR. Aortic valve replacement markedly reduced all-cause mortality compared to medical therapy (P < 0.001), with five-year crude mortality rates of 31.6% versus 49.3% and a difference in restricted mean survival time of 8.9 months. Similar outcomes were observed across high, intermediate, and low surgical risk categories. While TAVR demonstrated early mortality reduction compared to SAVR, no distinctions emerged in the overall five-year follow-up, regardless of surgical risk. Aortic valve replacement notably improved survival over conservative therapy. This study advocates for the preference of TAVR or SAVR in severe AS patients when feasible.

## Introduction and background

The rising prevalence of aortic stenosis (AS) represents a significant and escalating clinical challenge, affecting approximately 1.7% of the population aged 65 and over, with the incidence surging to 12% among those 75 years and older, of which 3.4% are classified with severe AS [1]. The management of symptomatic patients with severe AS has undergone significant evolution in recent years. Surgical aortic valve replacement (SAVR) has long been the cornerstone for the treatment of symptomatic severe AS, with recent guidelines expanding the indication for transcatheter aortic valve replacement (TAVR) to patients with symptomatic severe AS, ranging from those at prohibitive or high-risk to those at intermediate or even low risk for SAVR, informed by a series of clinical trials comparing TAVR and SAVR [2-8]. However, these trials have encountered challenges in assessing long-term mortality due to limitations in power, with many relying on composite outcomes and some prioritizing short-term all-cause mortality as the primary endpoint. Consequently, uncertainties persist regarding long-term mortality benefit comparison between patients undergoing TAVR and SAVR.

The efficacy of aortic valve replacement (AVR), whether through surgical or transcatheter approaches, in treating severe AS compared to conservative management has been documented in observational studies. Several small-scale clinical trials have demonstrated the superior outcomes of TAVR over standard treatment, particularly in patients deemed a prohibitive risk for SAVR [5] and a significantly lower incidence of all-cause death for early SAVR in asymptomatic patients with severe AS [9,10]. Recognizing the magnitude of this benefit is crucial for decision-making in managing patients with severe AS. Despite these insights, a knowledge gap persists regarding the clinical benefit of AVR compared to conservative management in real-world clinical settings with different patient risk profiles. 

In this study, we aimed to synthesize data from randomized controlled trials (RCTs) and non-RCTs to compare the outcomes of TAVR versus SAVR, as well as conservative management in patients with severe AS by pooling Kaplan-Meier-derived individual patient data (IPD) to standardize the inclusion criteria, scrutinize modeling assumptions, and directly model individual-level interactions within studies, thereby enhancing statistical power and mitigating confounding bias. We sought to bridge the knowledge gap by evaluating mortality benefits in severe AS patients across various risk profiles and management strategies.

This article was previously posted to the medRxiv preprint server on April 14, 2024.

## Review

Methods

This meta-analysis adhered to the principles delineated by the Preferred Reporting Items for Systematic Reviews and Meta-Analyses (PRISMA). The study was registered on the International Prospective Register of Systematic Reviews (PROSPERO) international prospective register of systematic reviews (CRD42024508950). Given its nature as a systematic review and meta-analysis, the study was considered exempt from local institutional research board review.

Strategy of Literature Search for the Meta-Analysis

We performed a systemic literature search for studies published in English after the year 2000, utilizing PubMed, Google Scholar, and Cochrane Library up to December 7, 2023. The search employed keywords pertaining to severe AS and AVR (Table 1). Additionally, we also screened reference lists of eligible original studies, systematic reviews, and meta-analyses to identify any other potentially eligible studies. Subsequently, full texts of the identified studies were retrieved and thoroughly reviewed. Studies published prior to 2000, as well as those categorized as reviews or meta-analyses, were excluded from consideration.

**Table 1:** Electronic database search strategy

Database	Search strategy	Search results
PubMed	("Transcatheter aortic valve replacement"[Title/Abstract] OR "transcatheter aortic valve implantation"[Title/Abstract]) OR TAVR[Title/Abstract]) OR TAVI[Title/Abstract] OR "surgical aortic valve replacement"[Title/Abstract] OR "surgical aortic valve replacement"[Other Term] OR "SAVR"[Title/Abstract]) AND ("outcome*"[Title/Abstract] OR "follow-up"[Title/Abstract]) AND ((“controlled study”[Publication Type] OR “multicenter study”[Publication Type] OR "randomized controlled trial"[Publication Type] OR "trial"[Title/Abstract])	1,504
	(“Severe Aortic Stenosis”[Title/Abstract]) AND ("Medical therapy”[Title/Abstract] OR "Conservative management"[Title/Abstract] OR "Natural history"[Title/Abstract] OR "Natural course"[Title/Abstract])	236
Google Scholar	("Transcatheter aortic valve replacement" OR "transcatheter aortic valve implantation" OR “TAVR” OR “TAVI” OR "surgical aortic valve replacement" OR "SAVR") ANA (“Severe Aortic Stenosis”) AND ("outcome" OR "follow-up") AND (“controlled study” OR “multicenter study” OR “randomized controlled trial”)	3,410
	(“Severe Aortic Stenosis”) AND ("Medical therapy” OR "Conservative management" OR "Natural history" OR "Natural course") AND ("outcome" OR "follow-up") AND (“All-cause mortality”)	4,720
Cochrane	("Transcatheter aortic valve replacement" OR "transcatheter aortic valve implantation" OR “TAVR” OR “TAVI” OR "surgical aortic valve replacement" OR "SAVR")	1,428
	(“Severe Aortic Stenosis”) AND ("Medical therapy” OR "Conservative management" OR "Natural history" OR "Natural course")	20

Data Extraction and Risk of Bias Assessment for the Meta-Analysis

Two independent reviewers systematically extracted the relevant data from each study, encompassing the first author, year of publication, study population characteristics (including left ventricular ejection fraction, mean aortic valve gradient, and aortic valve area), study size, study design, and country. Discrepancies were resolved by discussion. Raw data coordinates, comprising time, survival probability, or cumulative risks, along with the numbers at risk at specific time points and the total number of patients in each arm, were also extracted from the published Kaplan-Meier plots. The risk of bias in the included studies was assessed using version 2 of the Cochrane Risk of Bias tool for randomized trials (RoB) 2 or the Newcastle-Ottawa Scale (NOS) for cohort studies. 

*Study Selection* 

In the analysis comparing outcomes between TAVR and SAVR, we included studies in the meta-analysis that satisfied the following criteria: 1) RCTs; 2) propensity score-matched cohort studies derived from RCTs; 3) inclusion of graphed Kaplan-Meier curves depicting clinical outcomes in the text or appendix; and 4) a minimum of one-year follow-up for outcomes. For the comparison of outcomes between AVR and conservative management, we incorporated studies that met the following criteria: 1) RCTs specifically for TAVR and SAVR with reported all-cause mortality; 2) cohort studies comparing TAVR or SAVR to conservative management; 3) cohort studies presenting reported all-cause mortality for conservative management in severe AS; 4) inclusion of graphed Kaplan-Meier curves of all-cause mortality in the text or appendix; and 5) a minimum of one-year follow-up. 

Outcomes

The primary outcome of the meta-analysis was all-cause mortality at five years. Secondary endpoints included the composite of death from any cause, stroke, or myocardial infarction (MI). Additional secondary outcomes encompassed cardiovascular mortality and stroke or disabling stroke. The analysis also included an assessment of death from any cause, specifically in patients with paravalvular leaks following TAVR. The designated follow-up duration for all incorporated studies was truncated to five years.

Data Syntheses and Statistical Analysis

We reconstructed individual time-to-event data by pooling the extracted IPD using the two-stage modified iterative Kaplan-Meier approach [11]. We used the Kaplan-Meier Method to estimate the cumulative event rate for both primary and secondary endpoints, with analysis conducted through log-rank tests. Mixed effects Cox proportional hazards models were used to assess the mortality benefit. Models were fitted with a frailty term for study-level heterogeneity. The proportional hazards assumption of Cox models for each endpoint underwent verification using Schoenfeld residuals. In cases where the proportional hazards assumption was violated, two approaches were adopted. Firstly, we used landmark analysis, in which a landmark time was identified by visual inspection of the Schoenfeld residuals and Kaplan-Meier plots. The proportional hazards assumption was tested before and after the landmark time, and hazard ratios (HR) and 95% confidence intervals (CI) were calculated separately. Secondly, flexible hazard-based regression models were employed, utilizing a restricted cubic spline with internal knots located at six and 12 months for TAVR versus SAVR and at 12, 24, and 36 months for AVR versus conservative management. These models incorporated a time-varying treatment effect by involving an interaction term between the treatment effect and baseline hazard. For subgroup analysis, trials or cohort studies were stratified into three risk groups based on the Society of Thoracic Surgeons Predicted Risk of Mortality (STS PROM) (low risk, < 4%; intermediate risk, 4-8%; and high risk, > 8%). Studies lacking reported STS PROM were categorized based on cumulative event curves compared with other known studies. Sensitivity analyses were conducted, excluding non-clinical trials for TAVR versus SAVR or omitting studies without reported STS PROM. Additionally, the largest sample size study in the conservative management group was excluded from the AVR versus conservative management comparison. In a meta-analysis of mean values, the pooling used the inverse-variance method. For studies reporting median and interquartile range, mean and standard deviation were estimated based on the sample size, median, and first and third quartiles. To assess the clinical advantages of AVR in comparison to conservative management, the number needed to treat (NNT) and the restricted mean survival time (RMST) were computed. Results were presented as mean and 95% CI or ratio. The risk of outcome was quantified as HR and 95% CI. Statistical significance was determined at p-values < 0.05. All analyses were conducted with the R software, version 4.1.2 (The R Core Team, R Foundation for Statistical Computing, Vienna, Austria).

Results

Study Selection and Characteristics 

**Figure 1:**
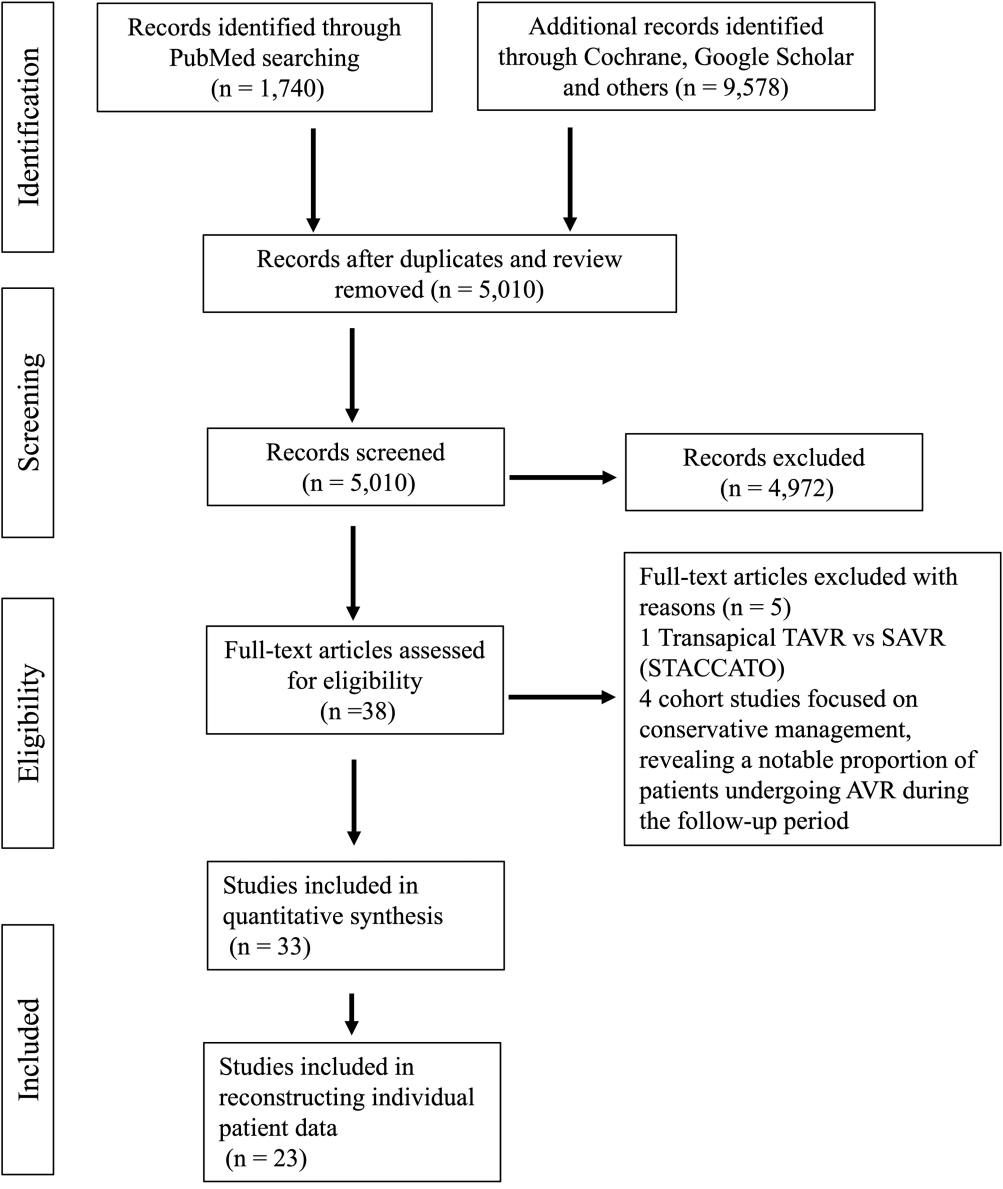
A PRISMA flowchart outlining the study selection process PRISMA: Preferred Reporting Items for Systematic Reviews and Meta-Analyses; TAVR: transcatheter aortic valve replacement; SAVR: surgical aortic valve replacement; STACCATO: A Prospective, Randomised Trial of Transapical Transcatheter Aortic Valve Implantation versus Surgical Aortic Valve Replacement in Operable Elderly Patients with Aortic Stenosis

**Figure 2:**
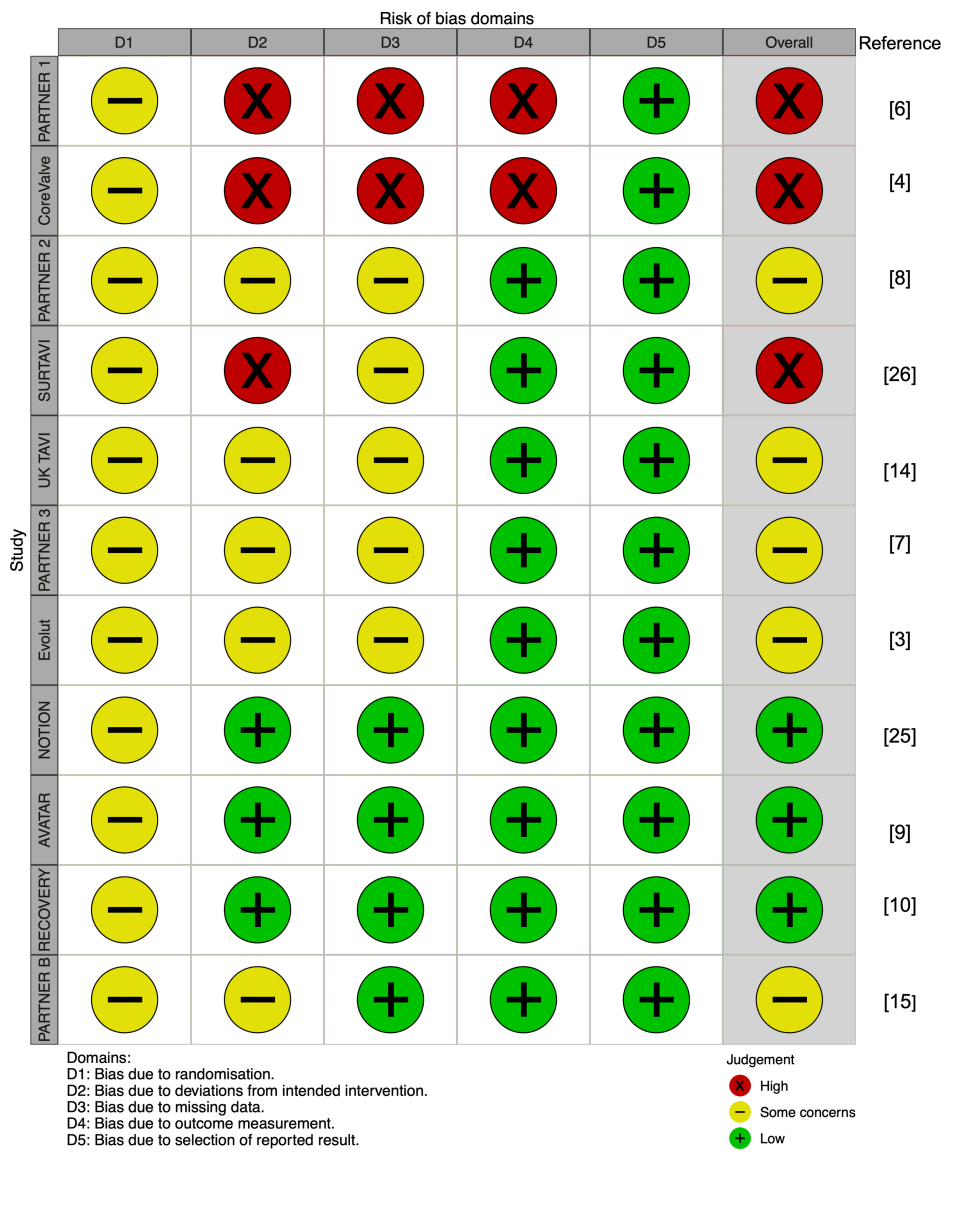
Risk of bias assessment for randomized trials Sources: [3, 4, 6, 7, 4, 8, 9, 10, 14, 15, 25, 26] PARTNER: Placement of Aortic Transcatheter Valves; SURTAVI: Surgical Replacement and Transcatheter Aortic Valve Implantation; UK TAVI: UK Transcatheter Aortic Valve Implantation; NOTION: Nordic Aortic Valve Intervention; AVATAR: Aortic Valve Replacement Versus Conservative Treatment in Asymptomatic Severe Aortic Stenosis; RECOVERY: Randomized Comparison of Early Surgery versus Conventional Treatment in Very Severe Aortic Stenosis

Following a careful review of studies based on predefined inclusion and exclusion criteria, a total of 23 clinical trials and retrospective cohort studies were identified for further data extraction and analysis [2-4, 6-10, 12-27]. The flowchart for the study selection is shown in Figure 1. The characteristics and quality assessment of the included studies are summarized in Table 2. Of these 23 studies, there were eight RCTs (CoreValve, Nordic Aortic Valve Intervention (NOTION), Evolut, Placement of Aortic Transcatheter Valves (PARTNER) 1, PARTNER 2, PARTNER 3, Surgical Replacement and Transcatheter Aortic Valve Implantation (SURTAVI), and UK Transcatheter Aortic Valve Implantation (UK-TAVI)) comparing TAVR with SAVR and three RCTs (Aortic Valve Replacement Versus Conservative Treatment in Asymptomatic Severe Aortic Stenosis (AVATAR), PARTNER B, and Randomized Comparison of Early Surgery versus Conventional Treatment in Very Severe Aortic Stenosis (RECOVERY)) comparing AVR with conservative management. The risk of bias for clinical trials was assessed in Figure 2. Baseline characteristics pooled from studies for the primary endpoint are detailed in Table 3. For TAVR (n = 4,215) and SAVR (n = 4,017), the mean age of patients was 79.5 years, with 41.1% and 44.0% being female, respectively. The mean STS-PROM scores for TAVR and SAVR were 4.8% and 4.9%, respectively. In the AVR and conservative management groups, the mean age was approximately 78.0 and 76.7, with 44.2% and 49.1% being female, respectively. The mean aortic valve area ranged from 0.72 to 0.77 cm^2^, and the mean aortic valve gradient was between 45.5 and 48.5 mmHg. 

**Table 2:** Characteristics of the included trials and studies RCT: randomized controlled trial; ROB2: a revised tool for assessing risk of bias in randomized trials; NOS: The Newcastle-Ottawa Scale; AVA: aortic valve area; AVR: aortic valve replacement; AS: aortic stenosis; Med Rx: medical therapy; PG: aortic pressure gradient; SAVR: surgical aortic valve replacement; TAVR: transcatheter aortic valve replacement; LVEF: left ventricular ejection fraction; PARTNER: Placement of Aortic Transcatheter Valves; SURTAVI: Surgical Replacement and Transcatheter Aortic Valve Implantation; UK TAVI: UK Transcatheter Aortic Valve Implantation; NOTION: Nordic Aortic Valve Intervention; AVATAR: Aortic Valve Replacement Versus Conservative Treatment in Asymptomatic Severe Aortic Stenosis; RECOVERY: Randomized Comparison of Early Surgery versus Conventional Treatment in Very Severe Aortic Stenosis

Study	Study acronym	Study design	Region	N	Mean age (years)	Follow-up (year)	Key criteria	Intervention	Primary endpoint	Risk of bias
Gleason et al., 2018 [4]	CoreValve	RCT	USA	797	83.2	5	Severe aortic stenosis and heart-failure symptoms of New York Heart Association (NYHA) class II or higher. Aortic stenosis was defined as AVA ≤0.8 cm^2^ (index 0.5 cm^2^/m^2^), peak velocity >4 m/s, or mean PG >40 mmHg. High surgical risk was defined as an estimated 30-day risk of surgical mortality and major morbidity of at least 15%, but <50%.	TAVR vs. SAVR	All-cause mortality at one year	ROB2: High
Forrest et al., 2023 [2]; Forrest et al., 2023 [3]	Evolut	RCT	Australia, Canada, France, Japan, the Netherlands, New Zealand, and the USA	1414	74	3 and 4	Severe aortic valve stenosis with trileaflet aortic valve morphology and a low predicted risk of death (<3%) from surgery. Severe, symptomatic AS AVA ≤ 1.0 cm^2^ (index 0.6 cm^2^/m^2^) with peak velocity ≥ 4 m/s or mean PG ≥ 40 mmHg.	TAVR vs SAVR	Composite of all-cause mortality or disabling stroke at 2 years	ROB2: Some concerns
Thyregod et al., 2019 [25]	NOTION	RCT	Denmark, Sweden	280	79.1	5	Severe, symptomatic AS AVA ≤1 m^2 ^(index 0.6 cm^2^/m^2^) and either peak velocity >4 m/s or mean PG >40 mmHg. Patients with acute treatment, severe coronary artery disease, severe non-aortic valvular disease, prior heart surgery, recent stroke or myocardial infarction (MI), or severe lung or renal disease were excluded.	TAVR vs. SAVR	Composite rate of all-cause mortality, stroke, or MI at one year and five years	ROB2: Low
Mack et al., 2015 [6]	PARTNER 1	RCT	USA, Canada, Germany	699	84.1	5	Severe, symptomatic AS AVA ≤0.8 cm^2^ (index 0.5 cm^2^/m^2^) or peak velocity ≥4 m/s or mean PG ≥40 mmHg. Patients were deemed to be at high risk for operative complications or death with an STS risk score of at least 10%.	TAVR vs. SAVR	All-cause mortality at one year	ROB2: High
Makkar et al., 2020 [8]	PARTNER 2	RCT	USA, Canada	2,032	81.6	5	Severe, symptomatic AS AVA ≤0.8 cm^2 ^(index 0.5 cm^2^/m^2^) or peak velocity >4 m/s or mean PG >40 mmHg. Patients were deemed to be at intermediate risk for operative complications or death with an STS risk score of 4-8%.	TAVR vs. SAVR	Composite of death from any cause or disabling stroke at two years	ROB2: Some concerns
Mack et al., 2023 [7]	PARTNER 3	RCT	USA, Australia, New Zealand, Japan	1,000	73.3	5	Severe, symptomatic AS AVA ≤1.0 cm^2^ (index 0.6 cm^2^/m^2^) with peak velocity ≥4 m/s or mean PG ≥40 mmHg. Asymptomatic if LVEF <50% or abnormal exercise test. Patients were deemed to be at intermediate risk for operative complications or death with an STS risk score of < 4%.	TAVR vs. SAVR	Composite of death from any cause, stroke, or rehospitalization at one year	ROB2: Some concerns
Van Mieghem et al., 2022 [26]	SURTAVI	RCT	USA, Canada, Germany, The Netherlands, UK, Spain, Switzerland, Sweden, Denmark	1,746	79.8	5	Severe, symptomatic AS AVA ≤1.0 cm^2^ (index 0.6 cm^2^/m^2^) with peak velocity >4 m/s or mean PG >40 mmHg or Doppler velocity index <0.25. Patients were deemed to be at intermediate risk for operative complications or death with an STS risk score of 3-15%.	TAVR vs. SAVR	Composite of death from any cause or disabling stroke at two years	ROB2: High
Toff et al., 2022 [14]	UK TAVI	RCT	UK	913	81	1	Severe, symptomatic AS Age ≥80 or ≥70 with intermediate or high-risk.	TAVR vs. SAVR	All-cause mortality at one year	ROB2: Some concerns
Banovic et al., 2022 [9]	AVATAR	RCT	Belgium, Czech Republic, Italy, Croatia, Lithuania, Poland, and Serbia	151	67	2	Patients >18 years old presenting with severe AS. Patients were excluded if they had exertional dyspnea, syncope or presyncope, angina, an LVEF <50%, severe AS (defined as maximal aortic jet velocity >5.5 m/s at rest), aortic regurgitation ≥3+, dilatation of the ascending aorta requiring replacement of aortic root or ascending aorta (>5 cm), or significant mitral valve disease, or if they had undergone previous cardiac surgery.	SAVR vs. Med Rx	All-cause mortality	ROB2: Low
Kang et al., 2020 [10]	RECOVERY	RCT	Korea	145	64	8	Patients who were 20 to 80 years of age and who presented with very severe aortic stenosis (an aortic-valve area of 0.75 cm^2^ or less with either a peak aortic jet velocity of 4.5 m per second or greater or a mean transaortic gradient of 50 mm Hg or greater). Patients were excluded if they had exertional dyspnea, syncope, presyncope, or angina, a left ventricular ejection fraction of less than 50%, clinically significant aortic regurgitation or mitral valve disease, or if they had undergone cardiac surgery.	SAVR vs. Med Rx	Composite of operative mortality or death from cardiovascular causes	ROB2: Low
Leon et al., 2010 [5]; Kapadia et al., 2014 [15]	PARTNER B	RCT	USA, Canada, Germany	449	TAVR: 83.0, Med Rx: 83.2	3	Symptomatic, mean gradient > 40 mm Hg or jet velocity > 4.0 m/s or an AVA of < 0.8 cm^2^ (or AVA index < 0.5 cm^2^/m^2^). Congenital unicuspid or congenital bicuspid valve was excluded.	TAVR vs. Med Rx	All-cause mortality at one year, two years, and three years	ROB2: Some concerns
Madhavan et al.,2023 [19]	SAPIEN 3	Propensity-score matched cohort	USA, Canada	1,566	81.6	5	Severe, symptomatic AS AVA ≤0.8 cm^2^ (index 0.5 cm^2^/m^2^) or peak velocity >4 m/s or mean PG >40 mmHg. Patients were deemed to be at intermediate risk for operative complications or death.	TAVR (PARTNER 2 S3 intermedia-te-risk (P2S3i) single-arm studies) vs SAVR (PARTNER 2)	Composite endpoint of death or disabling stroke	NOS: 8
Kvaslerud et al., 2021 [17]	Norway	Retrospective cohort	Norway	2,341	N/A	7	Age > 18 years, severe AS. Severe aortic stenosis was defined as an aortic valve area ≤ 1cm^2^, mean pressure gradient ≥ 40 mmHg, and maximal jet velocity ≥4m/s.	AVR vs. Med Rx	Seven-year survival	NOS: 7
Takeji et al., 2019 [23]	KC registry	Retrospective propensity-score matched cohort, registry	Japan	556	TAVR: 84.6, Med Rx: 85.1	2	Severe AS was defined as peak aortic jet velocity (Vmax) >4.0 m/s, mean aortic PG >40 mmHg, or AVA <1.0 cm^2^. Patients on hemodialysis and those asymptomatic patients with Vmax <5m/s and LVEF ≥ 50% were excluded.	TAVR vs. Med Rx	All-cause mortality	NOS: 7
Taniguchi et al., 2015 [24]	CURRENT registry	Retrospective propensity-score matched cohort, registry	Japan	582	AVR: 71.6, Med Rx: 73.1	5	Severe AS (peak aortic jet velocity (Vmax) >4.0 m/s, mean aortic PG >40 mm Hg, or AVA <1.0 cm^2^). Patients with a history of aortic valve repair/replacement/plasty or percutaneous aortic balloon valvuloplasty were excluded.	AVR vs. Med Rx	All-cause mortality	NOS: 9
Tourneau et al., 2010 [18]	Letour_STS	Retrospective cohort	USA	694	71	10	Patients aged 40 years or more diagnosed with asymptomatic severe AS are defined by a peak systolic velocity of 4 m/s or greater. Patients with multivalvular involvement, moderate to severe aortic regurgitation, history of clinical coronary artery disease, and prior aortic valve intervention or prior cardiac surgery of any cause were excluded.	SAVR vs. Med Rx	All-cause mortality	NOS: 7
Clark et al., 2012 [12]	US Medicare	Retrospective Cohort	USA	2,150	82	5	Patients who would be candidates for TAVR based on the presence of severe, symptomatic AS were considered to be at high risk for surgical AVR and were undergoing medical management.	Med Rx	Five-year survival	N/A
Généreux et al., 2023 [13]	Egnite	Retrospective cohort	USA	12,129	78.4	4	Patients with severe AS by echocardiographic reports and > 18 years of age.	Med Rx	All-cause mortality	N/A
Kitai et al., 2011 [16]	Kitai	Retrospective cohort	Japan	164	70	6	Patients with severe AS (a maximal jet velocity ≥ 4.0 m/s, or mean PG ≥ 40 mm Hg, or an AVA <1.0 cm^2^) and patients with very severe AS (a maximal jet velocity ≥ 5.0 m/s, or MPG ≥ 50 mm Hg or an AVA <0.6 cm^2^)	Med Rx	Six-year survival	N/A
Oh et al., 2019 [20]	Korean	Retrospective cohort	Korea	180	78	5	Symptomatic AS patients who refused to undertake AVR and were treated conservatively. Patients without symptoms, patients who underwent surgical or percutaneous AVR within a six-month interval from the diagnosis, patients with concomitant moderate to severe valvular diseases other than AS, and patients with other obvious causes of developing symptoms other than AS were excluded.	Med Rx	All-cause mortality	N/A
Strange et al., 2019 [21]	NEDA	Retrospective cohort, registry	Australia	6,383	N/A	15	Severe AS, characterized as either high-gradient (mean gradient >40.0 mm Hg and/or peak velocity >4.0 m/s with or without an AV area <1 cm^2^) or low-gradient (AV area <1 cm^2^ in the absence of high-gradient AS)	Med Rx	All-cause mortality	N/A
Suzuki et al., 2018 [22]	Suzuki	Retrospective cohort	Japan	63	87	4	Asymptomatic adults aged 80 and older with preserved left ventricular ejection fraction (LVEF > 50%) with severe AS ((AVA < 1.0 cm^2^).	Med Rx	All-cause mortality	N/A
Varadarajan et al., 2006 [27]	California	Retrospective cohort	USA	453	75	10	Severe aortic stenosis is defined as a valve area of 0.8 cm^2^ or less.	Med Rx	10-year survival	N/A

**Table 3:** Baseline characteristics for trials and studies comparing all-cause mortality. A Fib: atrial fibrillation; CABG: coronary artery bypass graft; CI: confidence interval; COPD: chronic obstructive pulmonary disease; Med Rx: medical conservative therapy; NYHA: New York Heart Association; PCI: percutaneous coronary intervention; SAVR: surgical aortic valve replacement; STS PROM: Society of Thoracic Surgeons Predicted Risk of Mortality; TAVR: transcatheter aortic valve replacement *Mean (95% CI) was derived from a meta-analysis of mean ± SD from the individual study.

	Aortic valve interventions		Interventions versus conservative therapy	
	TAVR (n=4215)	SAVR (n=4017)	AVR (n=11285)	Med Rx (n=23358)
Age, mean (95% CI) *, year	79.5 (76.3-82.8)	79.5 (76.2-82.9)	(n=9432) 78.0 (75.6-80.5)	(n=23084), 76.7 (73.2-80.2)
Female (%)	1732/4215 (41.1)	1767/4017 (44.0)	4172/9432 (44.2)	11330/23084 (49.1)
NYHA III or IV (%)	2172/3756 (57.8)	2051/3561 (57.6)	4427/7547 (58.7)	224/393 (57.0)
Creatinine>2.0 mg/dL (%)	69/2974 (2.3)	59/2779 (2.1)	186/6542 (2.8)	70/798 (8.8)
Diabetes (%)	1225/3867 (31.7)	1196/3663 (32.7)	2606/8510 (30.6)	3416/14156 (24.1)
Hypertension (%)	1781/2109 (84.4)	1729/2053 (84.2)	4130/5142 (80.3)	8593/14156 (60.7)
Peripheral vascular disease (%)	902/3748 (24.1)	859/3560 (24.1)	1896/8170 (23.2)	128/1050 (12.2)
Prior stroke (%)	116/1489 (7.8)	118/1400 (8.4)	294/3609 (8.1)	1176/13465 (8.7)
Cerebrovascular disease (%)	512/3612 (14.2)	485/3427 (14.2)	1056/7259 (14.5)	59/229 (25.8)
COPD (%)	1004/3724 (27.0)	966/3527 (27.4)	2111/7471 (28.3)	1626/14674 (11.1)
Home Oxygen (%)	127/1517 (8.4)	98/1480 (6.6)	275/3217 (8.5)	240/12358 (1.9)
Coronary artery disease (%)	1643/2976 (55.2)	1585/2827 (56.1)	3574/6194 (57.7)	4848/13678 (35.4)
Prior CABG (%)	654/4070 (16.1)	635/3882 (16.4)	1393/8741 (15.9)	192/12927 (1.5)
Prior PCI (%)	713/3371 (21.2)	662/3211 (20.6)	1530/7522 (20.3)	521/13078 (4.0)
Prior myocardial infarction (%)	379/3472 (10.9)	342/3305 (10.3)	788/7566 (10.4)	1160/13107 (8.9)
Pre-existing pacemaker/defibrillator (%)	406/4215 (9.6)	395/4016 (9.8)	849/8551 (9.9)	51/343 (14.9)
A Fib/A Flutter (%)	1099/4211 (26.1)	1044/4012 (26.0)	2293/9345 (24.5)	3176/14036 (22.6)
STS PROM, mean (95% CI), %	4.8 (2.0-7.6)	4.9 (2.1-7.7)	(n=9332), 4.8 (3.3-6.3)	(n=1629), 4.4 (1.4-7.5)
LVEF, mean (95% CI), %	(n=2241), 58.7 (55.2-62.2)	(n=1992), 58.4 (54.3-62.6)	(n=5292), 60.6 (57.9-63.2)	(n=20770), 61.3 (57.9-64.7)
Aortic valve pressure gradient, mean (95% CI), mmHg	(n=3531), 45.7 (43.2-48.2)	(n=3356), 45.5 (43.3-47.6)	(n=8046), 48.5 (45.8-51.2)	(n=20821), 45.5 (39.4-51.5)
Aortic valve area, mean (95% CI), cm^2^	(n=3419), 0.75 (0.68-0.81)	(n=3221), 0.77 (0.68-0.85)	(n=7639), 0.72 (0.68-0.76)	(n=20307), 0.74 (0.68-0.79)

All-Cause Mortality in TAVR Versus SAVR

**Figure 3:**
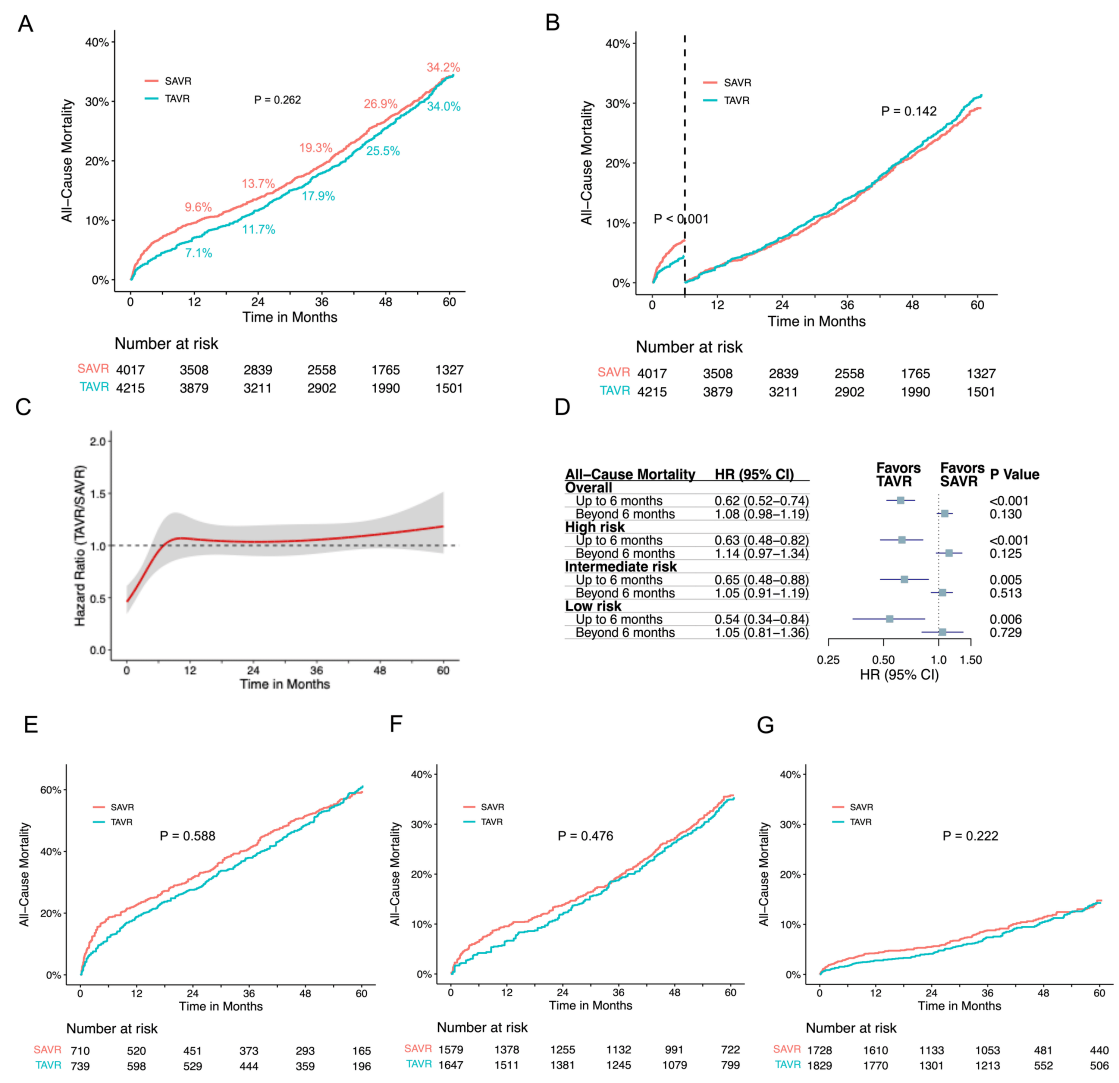
Time-to-event curves for all-cause mortality comparison between TAVR and SAVR (A) Pooled Kaplan-Meier plot illustrating reconstructed IPD analysis for all-cause mortality after transcatheter and surgical AVR; (B) Landmark analysis depicting all-cause mortality for TAVR versus SAVR; (C) Hazard ratios (HRs) with 95% confidence intervals depicting the dynamic change in all-cause mortality over time for TAVR compared to SAVR. The red curved line represents the time-varying hazard ratios, and the gray area denotes the 95% confidence interval; (D) Hazard ratios and corresponding 95% confidence intervals (CI) are presented for landmark analysis, both for the overall patient cohort and subgroups categorized by different risk profiles; (E) Pooled Kaplan-Meier plot illustrating all-cause mortality in high-risk patients; (F) Pooled Kaplan-Meier plot illustrating all-cause mortality in intermediate-risk patients; (G) Pooled Kaplan-Meier plot illustrating all-cause mortality in low-risk patients. AVR: aortic valve replacement; IPD: individual patient data; SAVR: surgical aortic valve replacement; TAVR: transcatheter aortic valve replacement

In this study, we analyzed seven RCTs (CoreValve, NOTION, Evolut, PARTNER 1, PARTNER 3, SURTAVI, and UK TAVI) and one cohort study (SAPIEN 3) to compare all-cause mortality in TAVR versus SAVR. As depicted in Figure 3A, the incidence rates of all-cause mortality were comparable between TAVR and SAVR (P = 0.262), with five-year crude mortality rates of 34.0% and 34.2%, respectively. Proportional hazard assumption violation was observed over the entire 60-month follow-up (P < 0.001). Time-varying hazard ratios (HRs) using flexible hazard models with restricted cubic spline indicated a relatively low HR for TAVR in the initial months compared to SAVR (Figure 3C). Landmark analyses with a six-month cutoff maintained proportional hazard assumptions. In the initial six months, TAVR demonstrated significantly lower all-cause mortality with an HR of 0.62 (95% CI: 0.52-0.74) compared to SAVR. In contrast, there was no difference in all-cause mortality beyond six months (HR 1.08, 95% CI: 0.98-1.19) (Figures 3B, 3D). 

**Figure 4:**
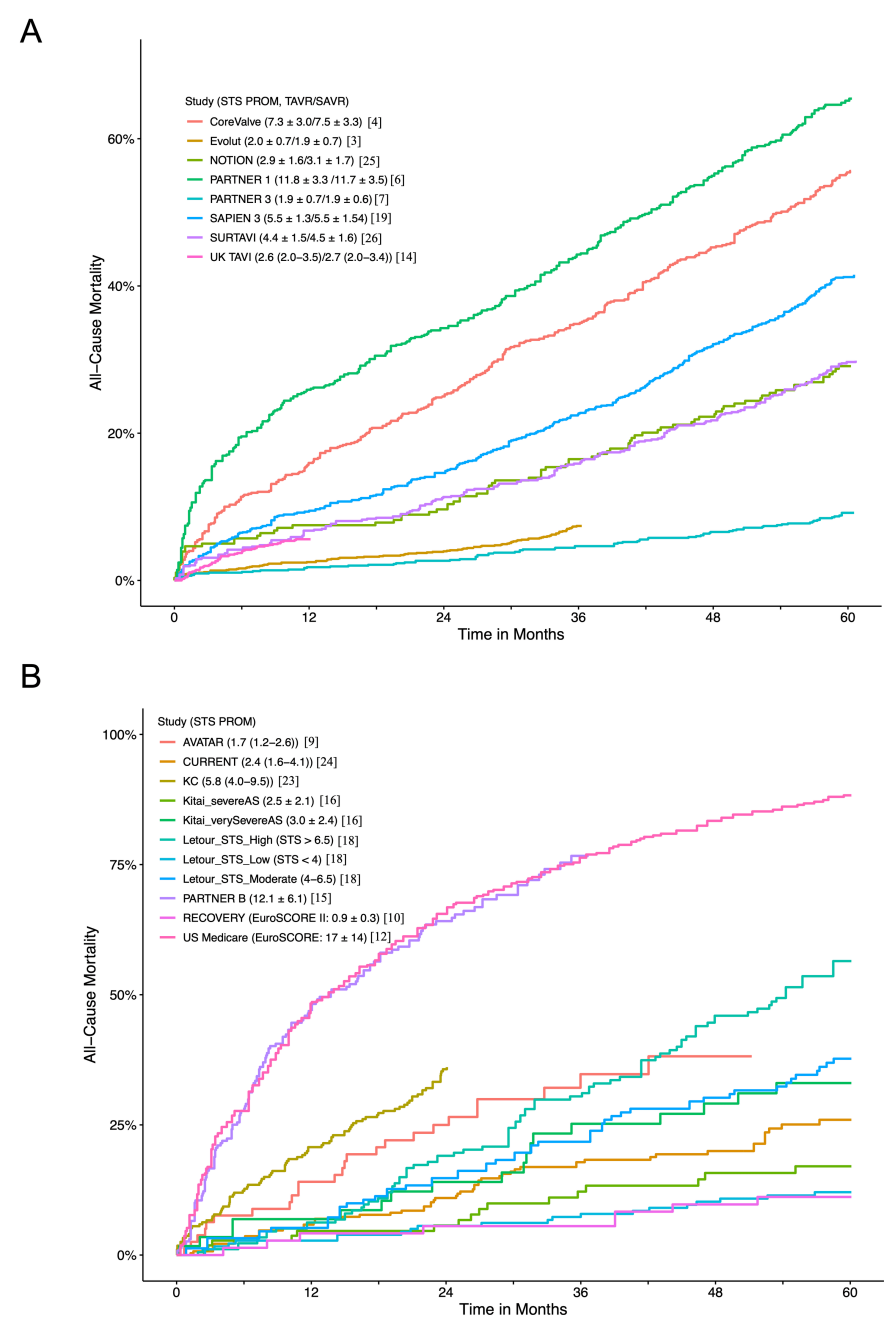
Correlation between STS PROM and all-cause mortality (A) Kaplan-Meier plot of all-cause mortality from individual studies encompassing both TAVR and SAVR. (B) Kaplan–Meier plot displaying the relationship between STS PROM and all-cause mortality in individual studies, specifically focusing on conservative management with reported STS PROM or EuroSCORE. STS PROM: Society of Thoracic Surgeons Predicted Risk of Mortality; PARTNER: Placement of Aortic Transcatheter Valves; SURTAVI: Surgical Replacement and Transcatheter Aortic Valve Implantation; UK TAVI: UK Transcatheter Aortic Valve Implantation; NOTION: Nordic Aortic Valve Intervention; AVATAR: Aortic Valve Replacement Versus Conservative Treatment in Asymptomatic Severe Aortic Stenosis; RECOVERY: Randomized Comparison of Early Surgery versus Conventional Treatment in Very Severe Aortic Stenosis; SAVR: surgical aortic valve replacement; TAVR: transcatheter aortic valve replacement; EUROScore: European System for Cardiac Operative Risk Evaluation

**Figure 5:**
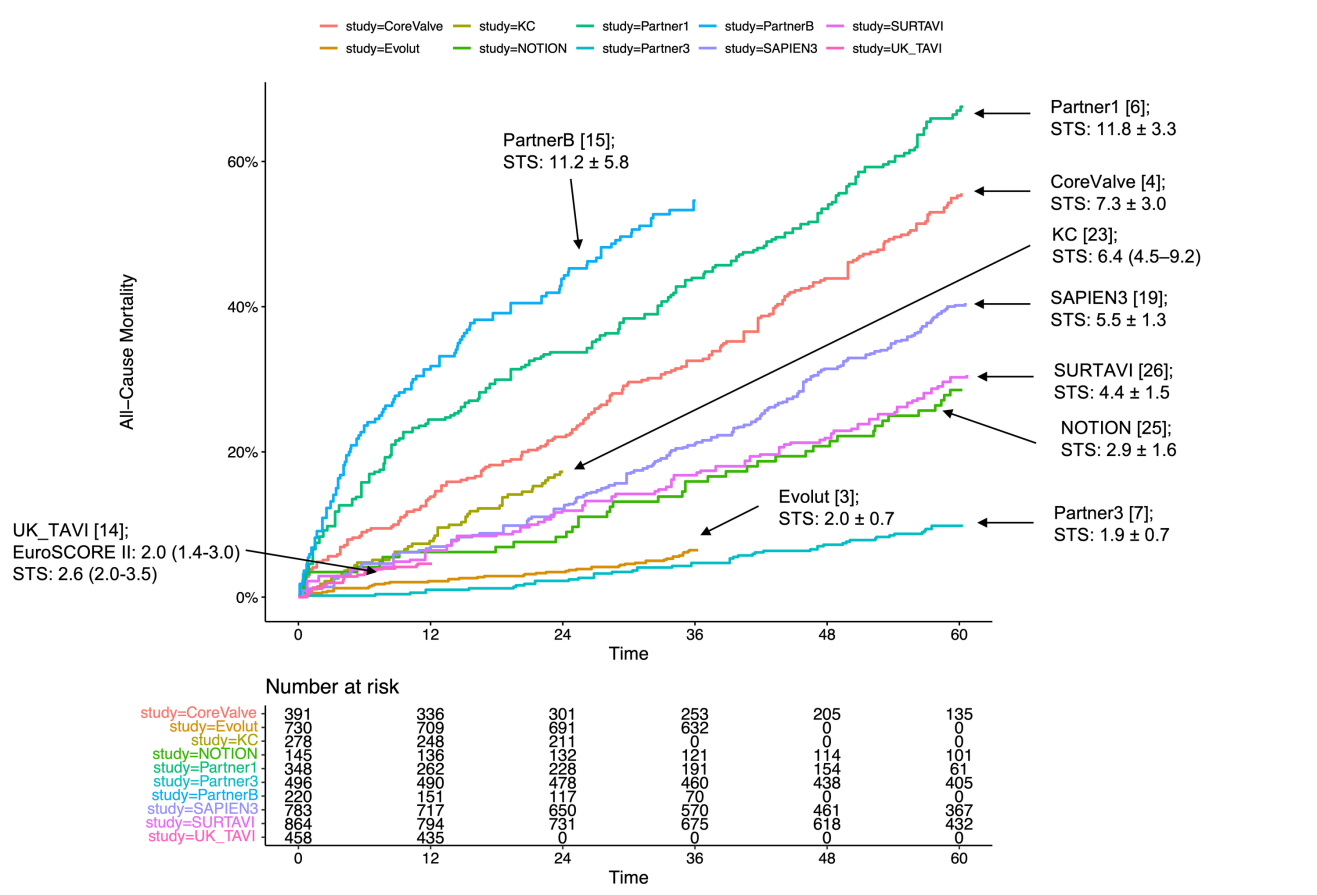
Robust correlation between the Society of Thoracic Surgeons Predicted Risk of Mortality (STS PROM) and all-cause mortality in patients undergoing TAVR. Each data point on the plot is labeled with its corresponding STS PROM if reported, emphasizing the alignment between mortality outcomes and the predicted risk scores. TAVR: transcatheter aortic valve replacement; PARTNER: Placement of Aortic Transcatheter Valves; SURTAVI: Surgical Replacement and Transcatheter Aortic Valve Implantation; UK TAVI: UK Transcatheter Aortic Valve Implantation; NOTION: Nordic Aortic Valve Intervention; AVATAR: Aortic Valve Replacement Versus Conservative Treatment in Asymptomatic Severe Aortic Stenosis; RECOVERY: Randomized Comparison of Early Surgery versus Conventional Treatment in Very Severe Aortic Stenosis; EUROScore: European System for Cardiac Operative Risk Evaluation

**Figure 6:**
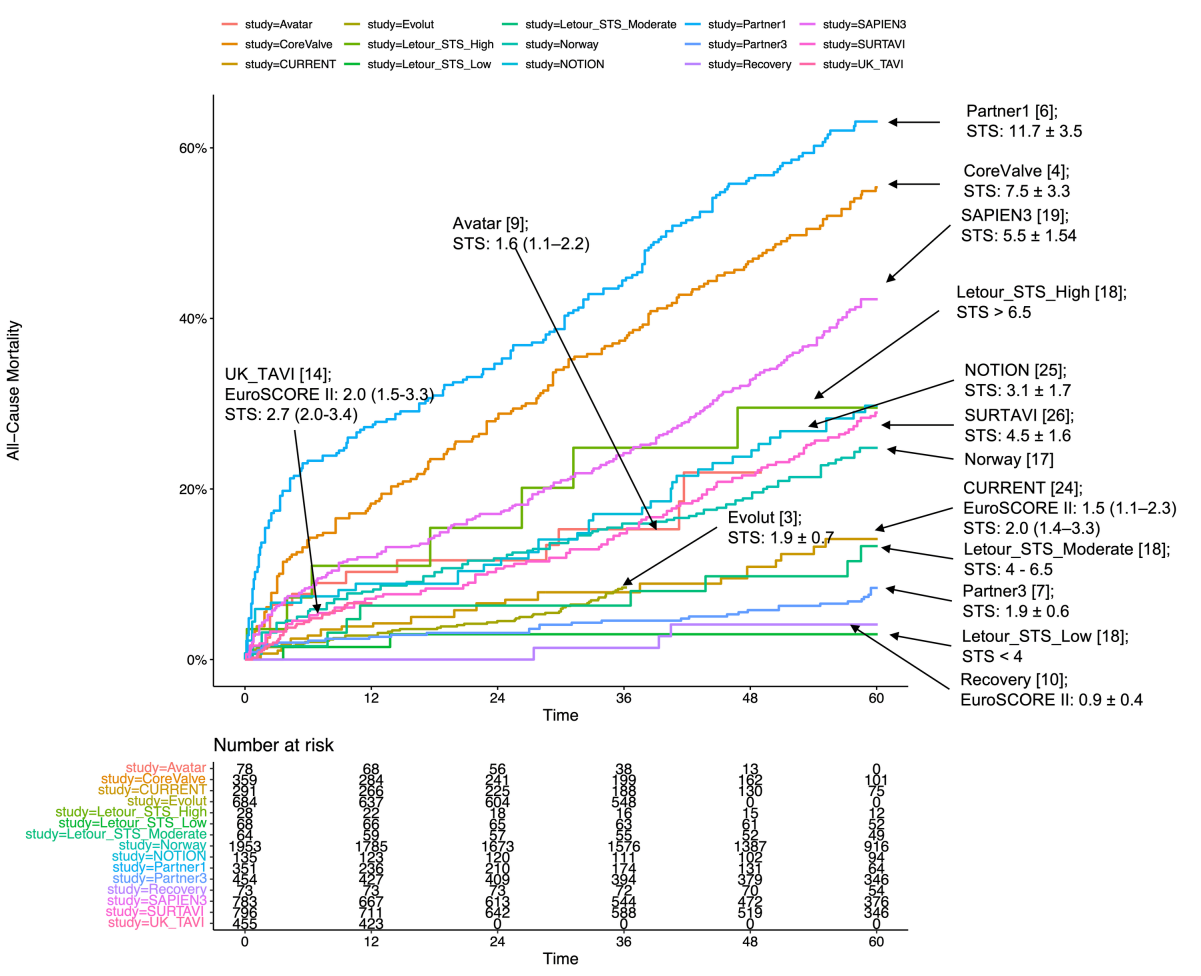
Robust correlation between the Society of Thoracic Surgeons Predicted Risk of Mortality (STS PROM) and all-cause mortality in patients undergoing SAVR Each data point on the plot is labeled with its corresponding STS PROM if reported, emphasizing the alignment between mortality outcomes and the predicted risk scores. SAVR: surgical aortic valve replacement; PARTNER: Placement of Aortic Transcatheter Valves; SURTAVI: Surgical Replacement and Transcatheter Aortic Valve Implantation; UK TAVI: UK Transcatheter Aortic Valve Implantation; NOTION: Nordic Aortic Valve Intervention; AVATAR: Aortic Valve Replacement Versus Conservative Treatment in Asymptomatic Severe Aortic Stenosis; RECOVERY: Randomized Comparison of Early Surgery versus Conventional Treatment in Very Severe Aortic Stenosis; EUROScore: European System for Cardiac Operative Risk Evaluation

**Figure 7:**
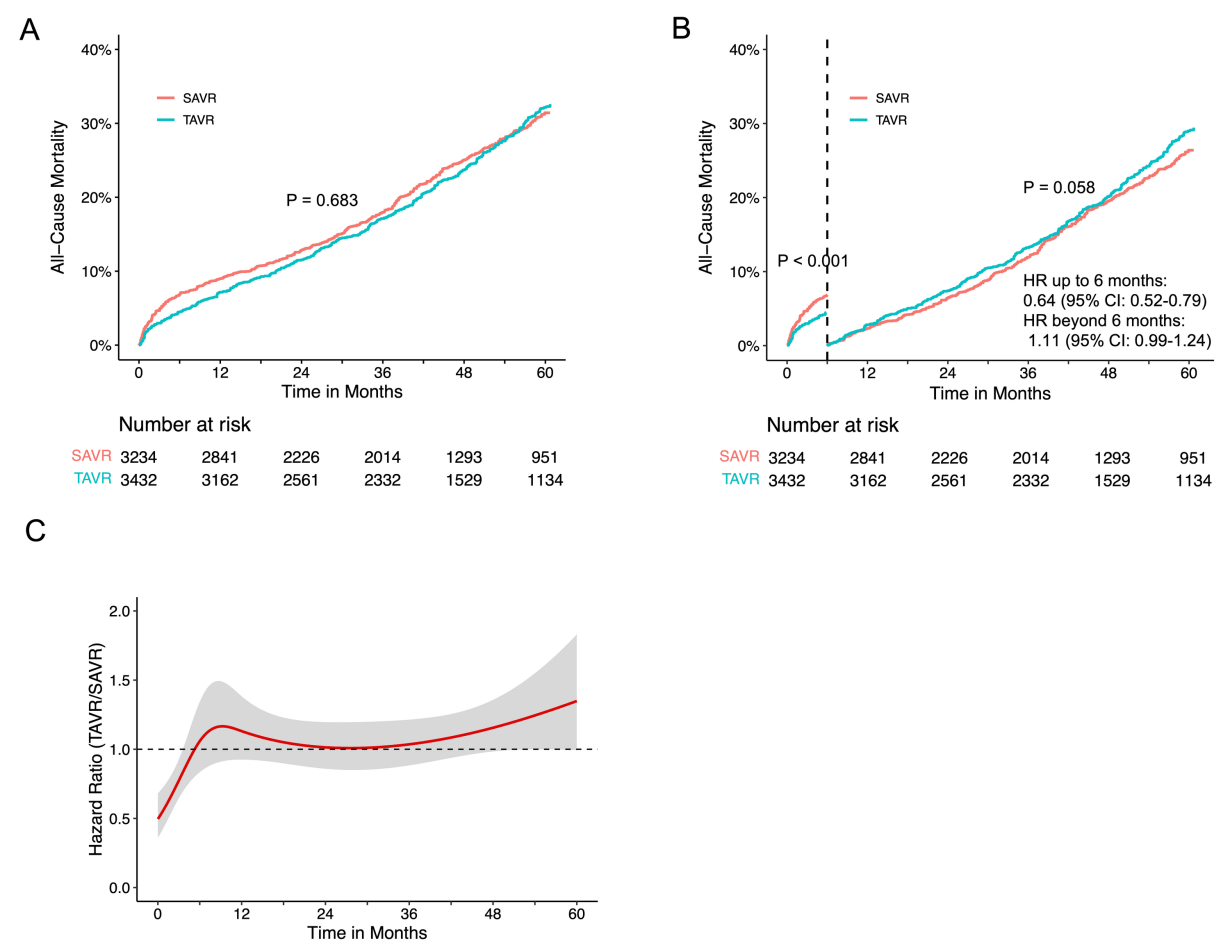
All-cause mortality for TAVR and SAVR in randomized trials (A) Pooled Kaplan-Meier plots illustrating all-cause mortality in randomized trials, excluding SAPIEN3 from Figure 1A. (B) Landmark analysis of all-cause mortality, comparing TAVR versus SAVR specifically within randomized trials. (C) Time-varying hazard ratios (HRs) with 95% confidence intervals, delineating the dynamic trends in all-cause mortality for TAVR versus SAVR within randomized trials. SAVR: surgical aortic valve replacement; TAVR: transcatheter aortic valve replacement

The correlation between STS PROM and five-year mortality for AVR, regardless of TAVR or SAVR, is illustrated in Figures 4A, 5, 6. Subgroup analyses based on STS PROM profiles were conducted to mitigate confounding due to varying risks. In the high-risk group (PARTNER 1 and CoreValve), no significant difference in all-cause mortality between TAVR and SAVR (P = 0.588) was observed over the five-year follow-up (Figure 3D, 3E), with proportional hazards assumption violation (P < 0.001). Transcatheter aortic valve replacement exhibited lower all-cause mortality in the initial six months (HR 0.63, 95% CI: 0.48-0.82), but this benefit diminished after six months (HR 1.14, 95% CI: 0.97-1.34). In the intermediate-risk group (SAPIEN 3 and SURTAVI), comparable all-cause mortality was noted between TAVR and SAVR (P = 0.476), with non-proportional hazards (P = 0.046). Transcatheter aortic valve replacement showed lower risk in the first six months (HR 0.65, 95% CI: 0.48-0.88), with similar risks beyond six months (HR 1.05, 95% CI: 0.91-1.19) (Figures 3D, 3F). The low-risk group (Evolut, PARTNER 3, UK TAVI, and NOTION) exhibited no significant difference (P = 0.222) in all-cause mortality between TAVR and SAVR over the five-year follow-up (HR 0.88, 95% CI: 0.71-1.10). In landmark analysis, TAVR demonstrated lower mortality risk up to six months (HR 0.54, 95% CI: 0.34-0.84) compared with SAVR, with similar risks after six months (HR 1.05, 95% CI: 0.81-1.36) (Figures 3D, 3G). Sensitivity analysis, excluding the cohort study SAPIEN 3 and only including RCTs, yielded similar results (Figure 7). 

All-Cause Mortality in AVR Versus Conservative Management

**Figure 8:**
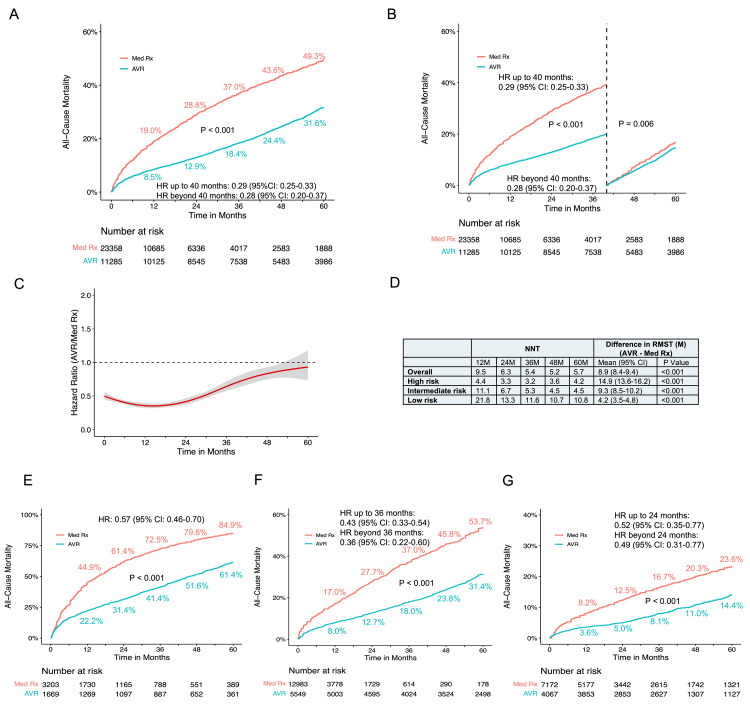
Time-to-event curves for all-cause mortality comparison between AVR and conservative management (A) Pooled Kaplan-Meier plot depicting reconstructed individual patient data (IPD) analysis for all-cause mortality post aortic valve replacement (AVR), contrasting with medical conservative management. (B) Landmark analysis depicting all-cause mortality for AVR versus conservative management. (C) Hazard ratios with 95% confidence intervals illustrate the time-varying dynamics of all-cause mortality for AVR compared to medical conservative management. The red curved line represents the time-varying hazard ratios, and the gray area denotes the 95% confidence interval. (D) The number needed to treat (NNT) and the difference in restricted mean survival time (RMST) are provided for the overall patient population and diverse risk subgroups. (E) Pooled Kaplan-Meier plot illustrating all-cause mortality in high-risk patients. (F) Pooled Kaplan-Meier plot illustrating all-cause mortality in intermediate-risk patients. (G) Pooled Kaplan-Meier plot illustrating all-cause mortality in low-risk patients. M: month; Med Rx: medical therapy

To investigate the benefits of AVR for treatment of severe AS, 10 RCTs (CoreValve, NOTION, Evolut, PARTNER 1, PARTNER 3, SURTAVI, UK TAVI, PARTNER B, AVATAR, and RECOVERY) and 12 cohort studies (SAPIEN 3, Norway, KC Registry, CURRENT, Letour_STS, US Medicare, Kitai, Korean, NEDA, Suzuki, California, and Egnite) were included for meta-analysis. The AVR significantly reduced all-cause mortality compared to conservative medical management (P < 0.001), with crude mortality rates at five years of 31.6% vs. 49.3% (Figure 8A). The NNT was 5.7 at five years and the RMST was 8.9 months greater with AVR than conservative management (P < 0.001) (Figure 8D). Proportional hazard assumption violation was evident over the entire 60-month follow-up (P < 0.001). Flexible hazard regression analysis showed a time-varying HR favoring AVR over conservative medical management throughout the entire follow-up period (Figure 8C). Landmark analyses with a 40-month cutoff maintained the proportional hazards assumption, revealing a lower risk of mortality for AVR with HRs of 0.29 (95% CI: 0.25-0.33) up to 40 months and 0.28 (95% CI: 0.20-0.37) beyond 40 months (Figure 8B).

**Figure 9:**
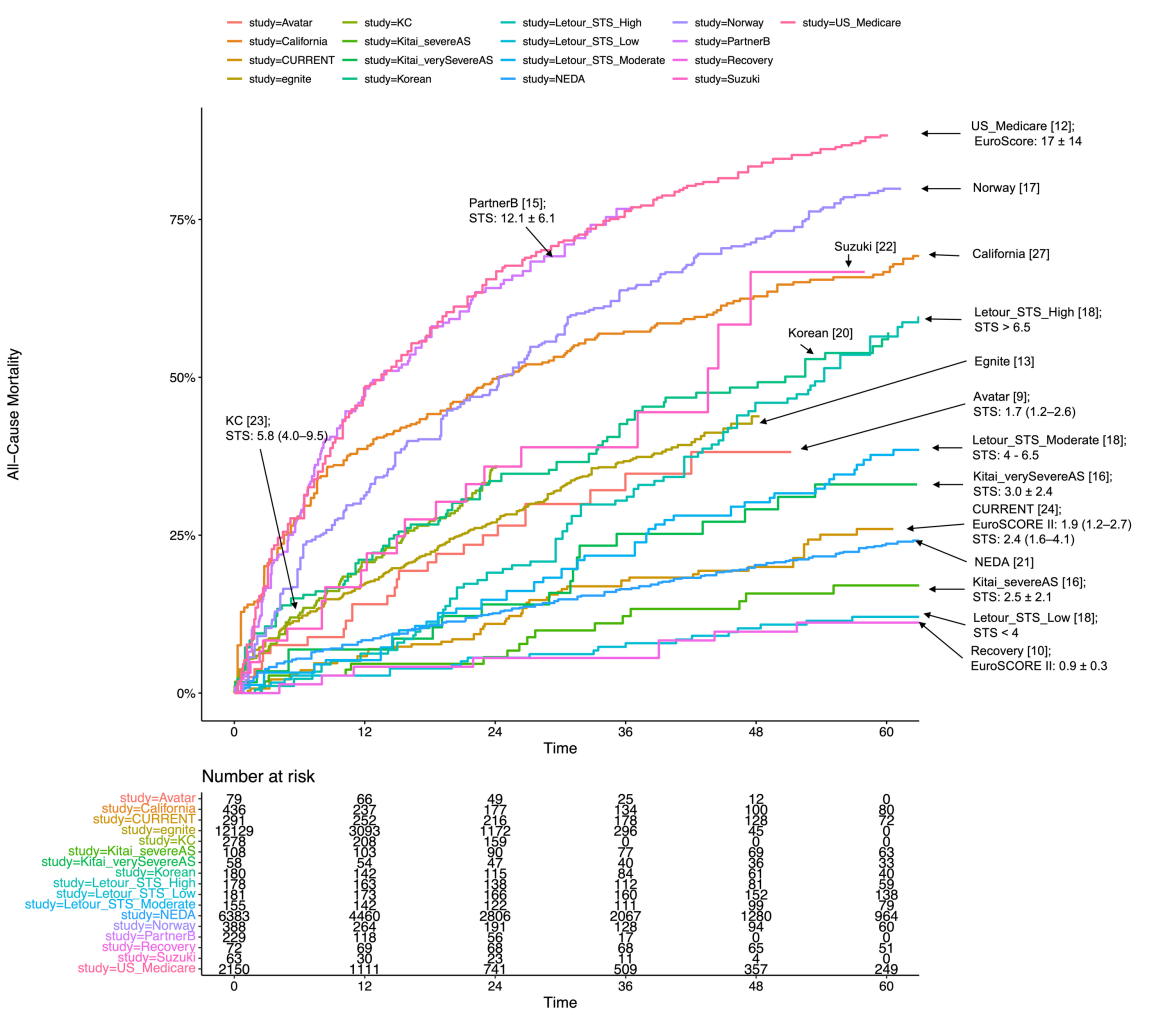
Correlation between the Society of Thoracic Surgeons Predicted Risk of Mortality (STS PROM) and all-cause mortality in patients undergoing conservative management Each data point on the plot is labeled with its corresponding STS PROM if reported, emphasizing the alignment between mortality outcomes and the predicted risk scores. EUROScore: European System for Cardiac Operative Risk Evaluation

**Figure 10:**
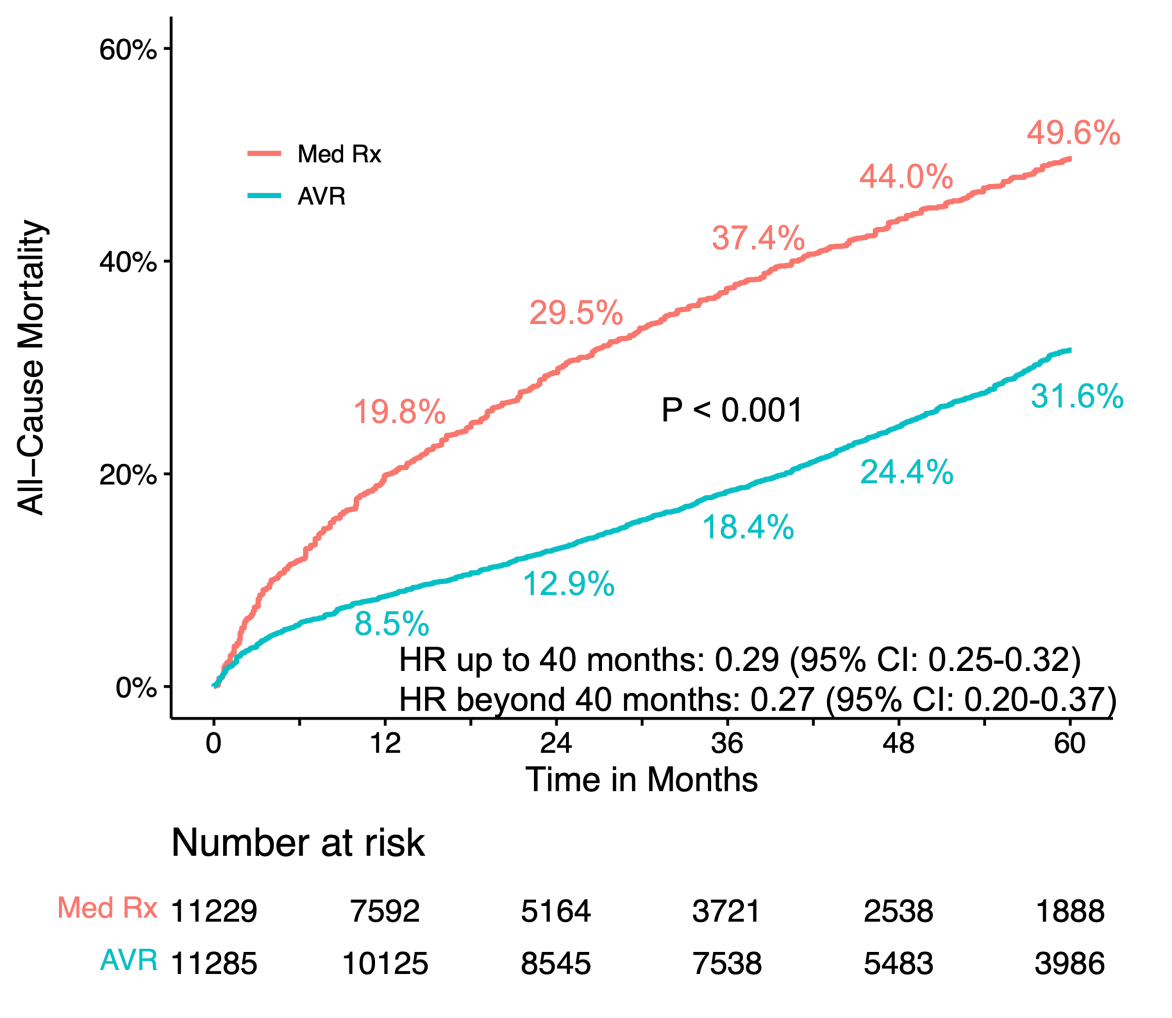
Sensitivity analysis of all-cause mortality for AVR versus medical conservative management excluding the Egnite study AVR: aortic valve replacement; Med Rx: medical therapy; HR: hazard ratio

**Figure 11:**
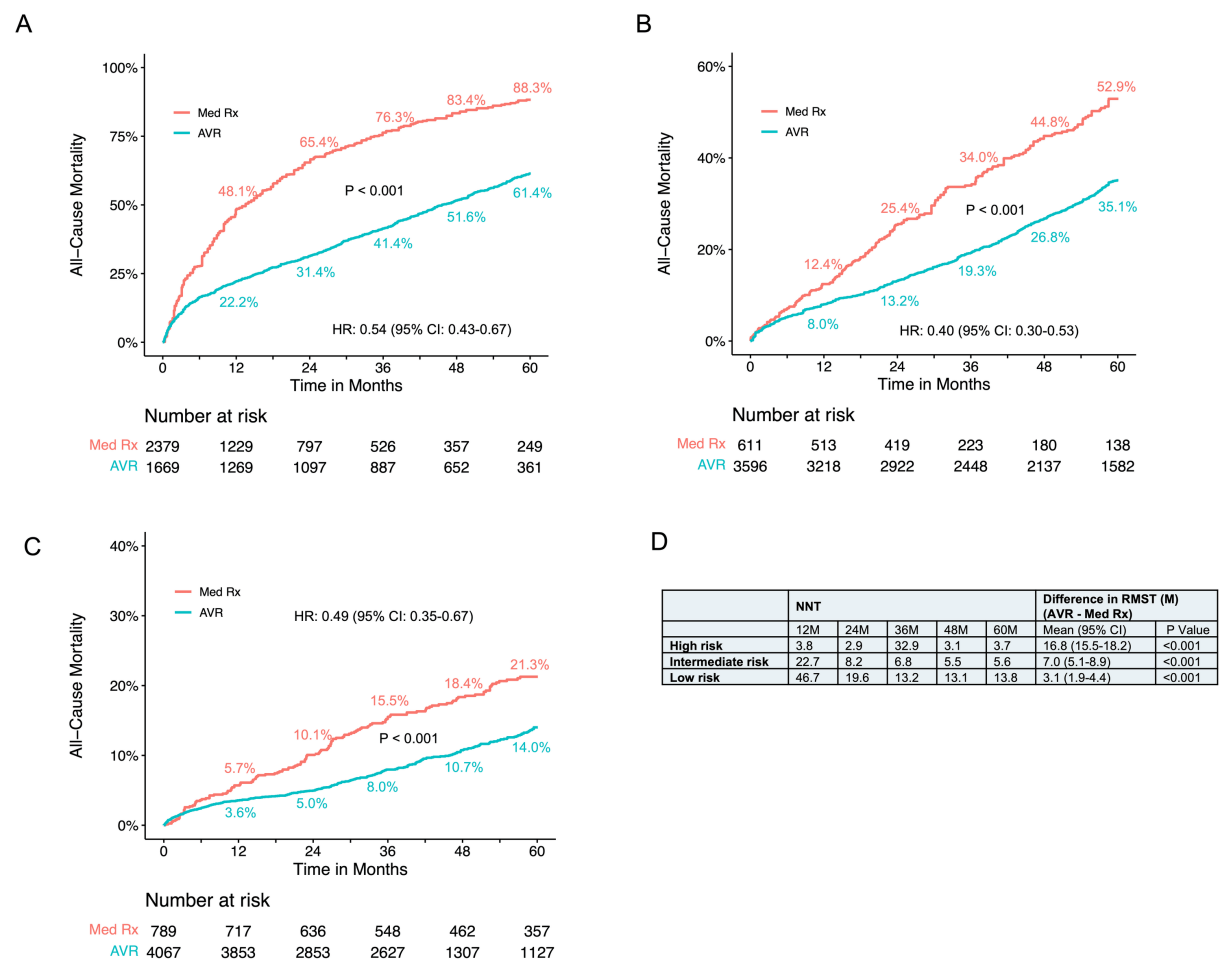
Sensitivity analysis of all-cause mortality for AVR versus medical conservative management in studies with reported STS PROM (A) Pooled Kaplan-Meier plot for all-cause mortality in high-risk patients. (B) Pooled Kaplan-Meier plot for all-cause mortality in intermediate-risk patients. (C) Pooled Kaplan-Meier plot for all-cause mortality in low-risk patients. (D) Number Needed to Treat (NNT) and the Difference in Restricted Mean Survival Time (RMST) across these different risk groups. AVR: aortic valve replacement; Med Rx: medical therapy; STS PROM: Society of Thoracic Surgeons Predicted Risk of Mortality

Subgroup analyses, considering the inherent heterogeneity in cohort studies, were conducted based on different STS PROM or European System for Cardiac Operative Risk Evaluation (EuroSCORE) scores. The correlation of STS PROM or EuroSCORE scores with long-term mortality for conservative medical therapy, except for AVATAR, was generally observed (Figures 4B, 9). In the high-risk group (PARTNER 1, CoreValve, US Medicare, Norway (conservative management arm), PARTNER B, California), crude mortality rates at five years were 61.4% for AVR and 84.9% for conservative management. The NNT was 4.2 at five years, and the RMST was 14.9 months greater with AVR (P < 0.001) (Figure 8D). There was a significant difference in all-cause mortality between AVR and conservative management (P < 0.001) during the five-year follow-up with an HR of 0.57 (95% CI: 0.46-0.70) (Figure 8E). In the intermediate-risk group (SAPIEN 3, SURTAVI, KC registry, Suzuki, Korean, Letour_STS, Norway (AVR arm)), crude mortality rates at five years were 31.4% for AVR and 53.7% for conservative management. The NNT was 4.5 at five years, and the RMST was 9.3 months greater with AVR (P < 0.001) (Figure 8D). The AVR was associated with a lower risk of all-cause mortality compared with conservative management (P < 0.001). Hazards changed non-proportionally over time in the intermediate-risk group (P = 0.040), with lower risk in AVR both in the first 36 months (HR 0.43, 95% CI: 0.33-0.54) and beyond 36 months (HR 0.36, 95% CI: 0.22-0.60) (Figure 8F). In the low-risk group (Evolut, PARTNER 3, UK TAVI, NOTION, Kitai, CURRENT, NEDA, Letour_STS, RECOVERY, AVATAR), crude mortality rates at five years were 14.4% for AVR and 23.6% for conservative management. The NNT was 10.8 at five years, and the RMST was 4.2 months greater with AVR (P < 0.001) (Figure 8D). There was a significantly lower risk of all-cause mortality for AVR throughout the five-year follow-up (log-rank P < 0.001) (Figure 8G). Proportional hazard assumption violation was observed (P < 0.001). In the landmark analysis, AVR was associated with a lower risk of mortality both in the first 24 months (HR 0.52, 95% CI: 0.35-0.77) and after 24 months with an HR of 0.49 (95% CI: 0.31-0.77) compared to conservative management (Figure 8G). Sensitivity analysis, excluding the largest study Egnite, or including only studies with reported STS PROM or EuroSCORE, yielded consistent results (Figures 10, 11).

*Secondary Endpoints* 

**Figure 12:**
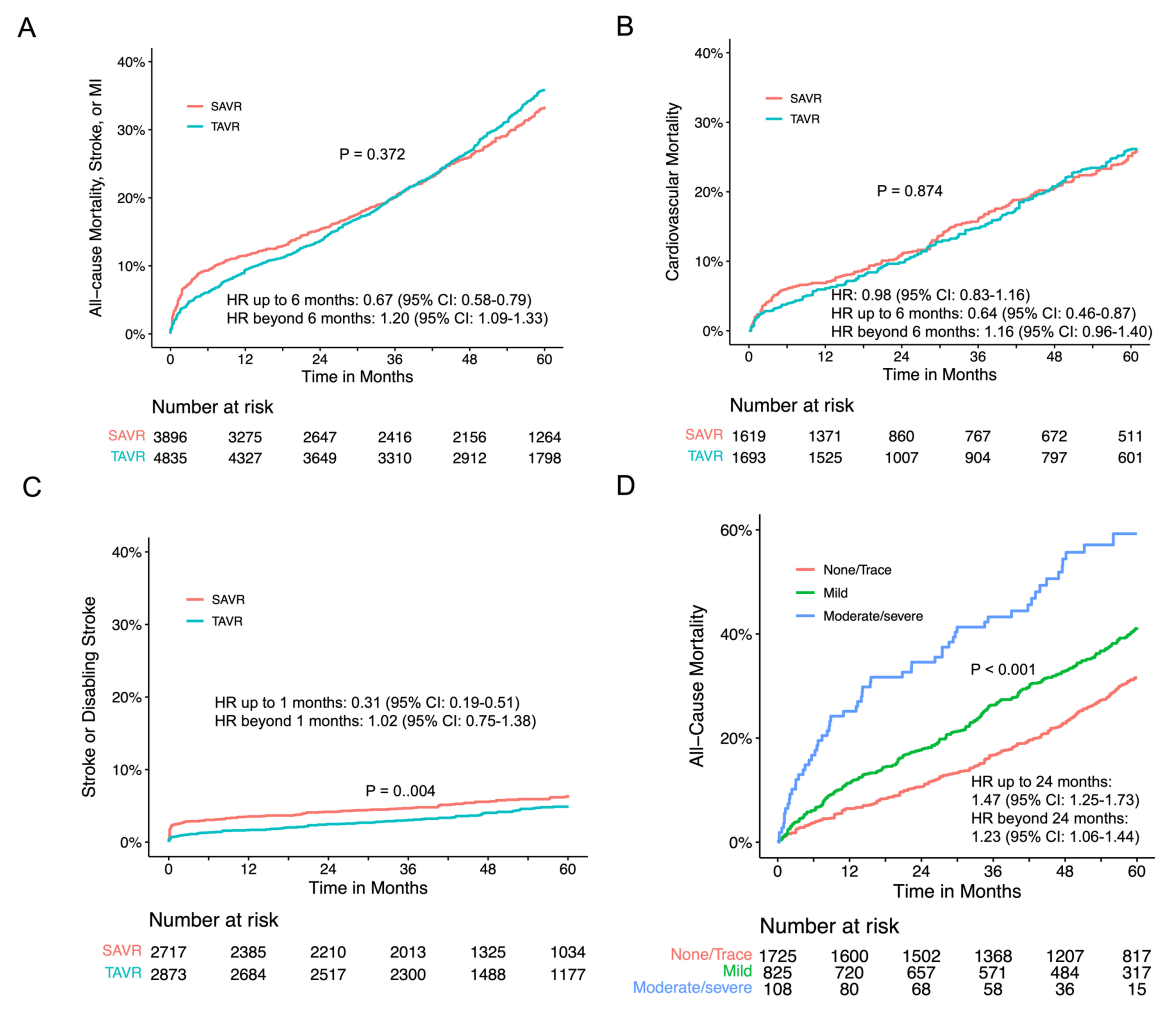
Secondary endpoints for TAVR and SAVR Pooled Kaplan-Meier plot illustrating the composite endpoint of all-cause mortality, stroke, or MI (A), cardiovascular mortality (B), stroke, or disabling stroke (C) following TAVR and SAVR. (D) Pooled Kaplan-Meier plot illustrating all-cause mortality stratified by the degree of paravalvular aortic regurgitation in patients undergoing TAVR. SAVR: surgical aortic valve replacement; TAVR: transcatheter aortic valve replacement; MI: myocardial infarction; HR: hazard ratio

**Figure 13:**
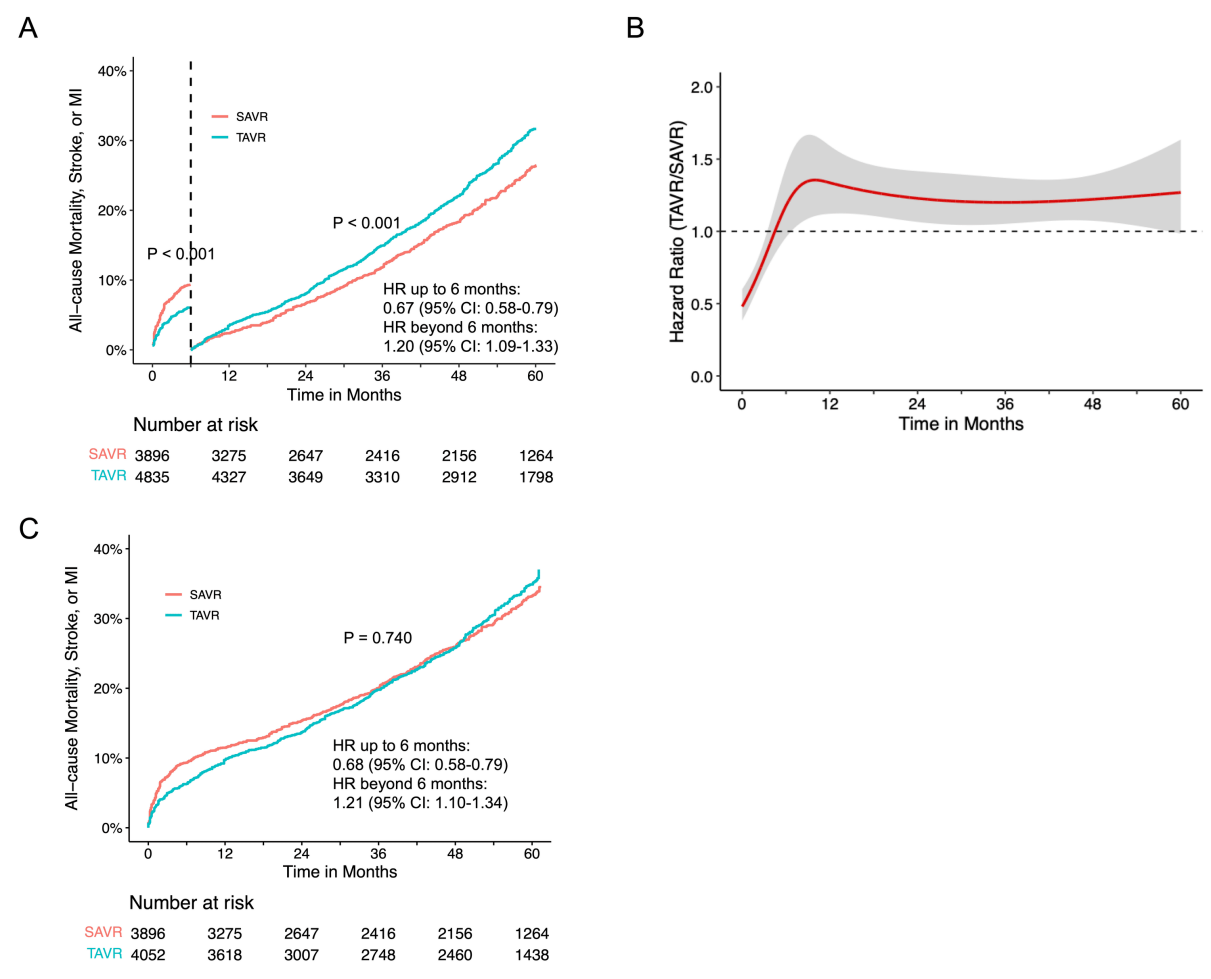
The composite of all-cause mortality, stroke or MI for TAVR versus SAVR. (A) Landmark analysis of the composite of all-cause mortality, stroke, or MI for TAVR versus SAVR. (B) Time-varying hazard ratios with 95% CIs for the composite of all-cause mortality, stroke, or MI for TAVR versus SAVR in randomized trials. (C) Pooled Kaplan-Meier plots for the composite endpoint in randomized trials, excluding SAPIEN3. MI: myocardial infarction; SAVR: surgical aortic valve replacement; TAVR: transcatheter aortic valve replacement

In the analysis of the composite of death from any cause, stroke, or MI, seven RCTs (NOTION, Evolut, PARTNER 1, PARTNER 2, PARTNER 3, SURTAVI, and UK TAVI) and the TAVR arm from SAPIEN3 were included. No difference in the composite endpoint between TAVR and SAVR (P = 0.372) was observed (Figure 12A). There was also evidence of proportional hazards assumption violation in the entire follow-up period (P < 0.001). Landmark analyses with a cutoff of six months maintained the proportional hazards assumption, revealing a lower risk of composite endpoint for TAVR with an HR of 0.67 (95% CI: 0.58-0.79) in the first six months. Conversely, beyond six months, TAVR demonstrated a higher risk for the composite endpoint (HR 1.20, 95% CI: 1.09-1.33) compared to SAVR (Figure 13A). This trend was consistently supported by flexible hazard regression analysis (Figure 13B). Sensitivity analysis, including only RCTs, produced similar results (Figure 13C).

For cardiovascular mortality, four RCTs (CoreValve, PARTNER 1, PARTNER 3, and UK TAVI) were included for meta-analysis. The incidence of cardiovascular mortality was comparable (P = 0.874) between TAVR and SAVR with an HR of 0.98 (95% CI: 0.83-1.16) (Figure 12B). Regarding stroke or disabling stroke, three studies (SURTAVI, SAPIEN3, and Evolut) reported the outcome of disabling stroke, and one RCT (PARTNER 3) reported stroke. Pooled analysis revealed that SAVR was associated with a higher rate of stroke or disabling stroke (P = 0.004). Landmark analysis revealed a high risk of stroke or disabling stroke in the first month (HR 0.31, 95% CI: 0.19-0.51) and a similar incidence of stroke after 1 month (HR 1.02, 95% CI: 0.75-1.38) (Figure 12C). 

For all-cause mortality in patients with paravalvular regurgitation, a meta-analysis included five RCTs (PATNER 1, PATNER 2, PATNER 3, PATNER B, and SURTAVI). A significant difference was observed among the various severity levels of paravalvular regurgitation (P < 0.001) (Figure 12D). The proportional hazards assumption did not hold during the follow-up period (P = 0.013). Landmark analysis revealed an increased risk of all-cause mortality in patients with increased severity levels of paravalvular regurgitation with an HR of 1.47 (95% CI: 1.25-1.73) in the first 24 months and an HR of 1.23 (95% CI: 1.06-1.44) beyond 24 months (Figure 12D).

Hemodynamics 

**Figure 14:**
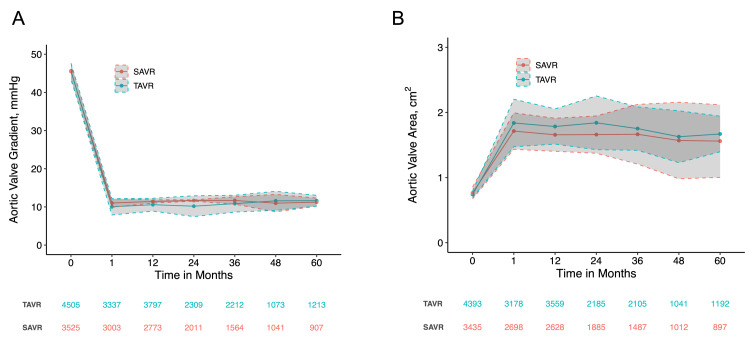
Long-term hemodynamic meta-analysis of aortic valve gradient and orifice area in transcatheter versus surgical patients over five years (A) Weighted mean with solid lines and 95% confidence intervals with dotted lines for aortic valve pressure gradient; (B) Weighted mean with solid lines and 95% confidence intervals with dotted lines for aortic valve orifice area SAVR: surgical aortic valve replacement; TAVR: transcatheter aortic valve replacement

Six RCTs (Evolut, PARTNER 1, PARTNER 2, PARTNER 3, SURTAVI, and UK TAVI) provided data on mean aortic valve area and pressure gradient measured by serial echocardiograms pre- and post-operation. In addition, the SAPIEN3 cohort study, specifically from its TAVR arm, has contributed measurements of the mean aortic valve area. Before the intervention, the mean aortic valve effective orifice area was 0.74 cm^2^ (95% CI: 0.69-0.79) for TAVR and 0.76 cm^2^ (95% CI: 0.67-0.85) for SAVR. At one month post-operatively, the mean aortic valve effective orifice area for TAVR and SAVR were 1.84 cm^2^ (95% CI: 1.47-2.20) and 1.71 cm^2^ (95% CI: 1.43-1.99), respectively. The mean effective orifice area appeared relatively stable over the 5-year follow-up period (Figure 14A). Preoperative mean aortic valve pressure gradients were 45.6 mmHg (95% CI: 43.6-47.6) for TAVR and 45.5 mmHg (95% CI: 43.3-47.6) for SAVR. At one month, these values decreased to 10.0 mmHg (95% CI: 7.9-12.2) and 11.0 mmHg (95% CI: 9.9-12.1), respectively. Similarly, the mean pressure gradient remained consistent throughout the five-year follow-up period (Figure 14B).

Discussion

Surgical aortic valve replacement has long been the cornerstone for the treatment of symptomatic severe AS. Over the last decade, TAVR has emerged as a viable alternative, even for patients at low risk of operative mortality. However, determining the superior treatment modality for severe AS remains inconclusive. In this meta-analysis involving patients with severe AS and utilizing Kaplan-Meier-derived IPD from RCTs, we observed no significant difference in all-cause mortality between TAVR and SAVR over the five-year follow-up period, irrespective of operative mortality risk profiles. Notably, TAVR exhibited a significantly lower risk of all-cause death in the initial six months. Additionally, by synthesizing data from both RCTs and cohort studies, our analysis revealed a notable association between AVR and lower risk for all-cause death.

Evidence has consistently shown that the STS PROM score is a robust predictor. It has been documented that STS PROM not only forecasts 30-day mortality but also extends its predictive accuracy to long-term mortality after cardiac surgical procedures [28]. In alignment with this, our study illustrates a robust correlation between STS PROM and five-year mortality for patients undergoing SAVR, as well as those opting for TAVR. Additionally, the EuroSCORE II, a metric comparable to STS PROM in predicting operative mortality, demonstrates superior accuracy compared to EuroSCORE I, which tends to overestimate operative mortality [29]. Based on these, we present the first demonstration that STS PROM and EuroSCORE can also effectively predict five-year mortality in severe AS patients undergoing conservative management. Building upon these insights, we proposed that these risk scores can be extrapolated from survival curves in studies lacking reported STS PROM, facilitating the categorization of patients into subgroups to mitigate heterogeneities and biases. Our sensitivity analyses validated the robustness and consistency of these findings across studies, regardless of STS PROM reporting status.

Aligned with previously published data, the proportional hazards assumptions for all-cause mortality in overall, high, and intermediate-risk groups were found to be violated. Employing STS PROM, our analysis revealed no significant differences in all-cause mortality between TAVR and SAVR in high- and intermediate-risk groups over the follow-up period. Notably, TAVR exhibited superiority in the initial six months, corroborating findings reported in high-risk groups, including both high- and intermediate-risk individuals, stratified according to STS PROM [30]. In the low-risk group, leveraging extended follow-up data compared to previous studies, our study yielded consistent results like those observed in the high and intermediate-risk groups. Considering the limited data on the mortality burden for severe AS in the modern era and the associated mortality benefits after AVR, our systematic analysis robustly demonstrated a significant association between AVR and mortality benefits. This effect was particularly pronounced in high- and intermediate-risk groups, characterized by a low NNT and prolonged survival gain. Even in low-risk groups, encompassing asymptomatic severe AS cases, our synthesized data advocates for AVR over conservative management for mortality benefits. 

Our study has several inherent limitations. Firstly, the inclusion of data synthesized from different generations of transaortic valves, employing varied deployment strategies, may not fully capture the current landscape of clinical practice. Secondly, the use of IPD derived from Kaplan-Meier plots, while considered a well-accepted approach, may introduce some compromise in data quality compared to a true IPD meta-analysis. Thirdly, the diverse outcomes reported across trials, coupled with variations in definitions and follow-up durations, could introduce selection and measurement bias when amalgamating data for meta-analysis. Although we attempted to address study-level heterogeneity through appropriate modeling, the potential for bias persists. Fourthly, the paucity of RCTs directly comparing AVR to conservative medical management led to the inclusion of many cohort studies. Despite efforts to mitigate confounding factors by stratification using STS PROM, the retrospective nature of these studies introduces inherent limitations, and residual confounders and bias may persist. Lastly, the generalizability of our findings may be limited, as they might not be readily extrapolated to patient populations excluded from the trials, such as those with bicuspid aortic valves, preexisting bioprosthetic or mechanical heart valves, and younger individuals. These exclusions underscore the importance of interpreting our results within the specified study population parameters.

## Conclusions

This meta-analysis, utilizing Kaplan-Meier-derived IPD, offers valuable insights into the comparative outcomes of TAVR compared to SAVR and conservative management compared with AVR for severe AS. Our findings reveal no significant disparities in all-cause mortality and cardiovascular mortality over a five-year period between TAVR and SAVR. However, TAVR demonstrated a noteworthy advantage with a notably lower risk of all-cause and cardiovascular mortality in the initial six months. Furthermore, TAVR exhibited a reduced risk of stroke compared to SAVR during the first month post procedure. The mean aortic valve area and pressure gradient remained comparable between TAVR and SAVR, exhibiting stability throughout the five-year follow-up. Additionally, AVR was associated with a considerable mortality benefit when compared to conservative management, regardless of risk profile. This comprehensive analysis not only contributes to our understanding of mortality outcomes for severe AS patients across different risk categories and treatment approaches but also holds significant implications for informing clinical decision-making and shaping policies in the field of structural cardiology.
